# A systematic review of type 2 diabetes mellitus and hypertension in imaging studies of cognitive aging: time to establish new norms

**DOI:** 10.3389/fnagi.2014.00148

**Published:** 2014-07-08

**Authors:** Liesel-Ann C. Meusel, Nisha Kansal, Ekaterina Tchistiakova, William Yuen, Bradley J. MacIntosh, Carol E. Greenwood, Nicole D. Anderson

**Affiliations:** ^1^Baycrest Centre, Rotman Research InstituteToronto, ON, Canada; ^2^Sunnybrook Research Institute, Heart and Stroke Foundation Canadian Partnership for Stroke RecoveryToronto, ON, Canada; ^3^Department of Medical Biophysics, Faculty of Medicine, University of TorontoToronto, ON, Canada; ^4^Department of Nutritional Sciences, Faculty of Medicine, University of TorontoToronto, ON, Canada; ^5^Departments of Psychology and Psychiatry, University of TorontoToronto, ON, Canada

**Keywords:** type 2 diabetes mellitus, hypertension, cognition, aging, imaging

## Abstract

The rising prevalence of type 2 diabetes (T2DM) and hypertension in older adults, and the deleterious effect of these conditions on cerebrovascular and brain health, is creating a growing discrepancy between the “typical” cognitive aging trajectory and a “healthy” cognitive aging trajectory. These changing health demographics make T2DM and hypertension important topics of study in their own right, and warrant attention from the perspective of cognitive aging neuroimaging research. Specifically, interpretation of individual or group differences in blood oxygenation level dependent magnetic resonance imaging (BOLD MRI) or positron emission tomography (PET H_2_O^15^) signals as reflective of differences in neural activation underlying a cognitive operation of interest requires assumptions of intact vascular health amongst the study participants. Without adequate screening, inclusion of individuals with T2DM or hypertension in “healthy” samples may introduce unwanted variability and bias to brain and/or cognitive measures, and increase potential for error. We conducted a systematic review of the cognitive aging neuroimaging literature to document the extent to which researchers account for these conditions. Of the 232 studies selected for review, few explicitly excluded individuals with T2DM (9%) or hypertension (13%). A large portion had exclusion criteria that made it difficult to determine whether T2DM or hypertension were excluded (44 and 37%), and many did not mention any selection criteria related to T2DM or hypertension (34 and 22%). Of all the surveyed studies, only 29% acknowledged or addressed the potential influence of intersubject vascular variability on the measured BOLD or PET signals. To reinforce the notion that individuals with T2DM and hypertension should not be overlooked as a potential source of bias, we also provide an overview of metabolic and vascular changes associated with T2DM and hypertension, as they relate to cerebrovascular and brain health.

## Introduction

Amongst middle-aged and older adults, the rising prevalence of T2DM, hypertension, and other conditions that comprise the metabolic syndrome is a global health epidemic, attributed largely to sedentary lifestyles, poor diet, and lack of exercise. In 2008, it was estimated that 347 million adults worldwide had T2DM, up from 153 million in 1980 (Danaei et al., 2011). Over the next two decades, it is expected that these numbers will continue to rise, by as much as 38% by 2030 (Shaw et al., 2010). Prevalence rates of hypertension are even higher. In 2000, the global prevalence of hypertension was 26.4%, affecting an estimated 972 million people worldwide. Again, these numbers are expected to increase by approximately 60% by 2025, to a total of 1.56 billion people (Kearney et al., 2005). Critically, hypertension is present in up to 75% of individuals with T2DM (Colosia et al., 2013). The growing number of middle-aged and older adults living with T2DM and/or hypertension makes these conditions important topics of study in their own right.

Better long-term health care and disease management allow middle-aged and older adults to live with T2DM and hypertension for many years; however, both of these conditions have long-term deleterious effects on cerebrovascular and brain health, and contribute to cognitive impairment and decline (Gorelick et al., 2011). T2DM and midlife hypertension confer a high risk for development of mild cognitive impairment (MCI) and dementia (Launer et al., 2000; Kloppenborg et al., 2008; Creavin et al., 2012; Crane et al., 2013; Roberts et al., 2014), and older individuals with T2DM progress to dementia at faster rates (Xu et al., 2010; Morris et al., 2014). These changing health demographics have created a discrepancy: what we define as “normal” or “typical” cognitive aging is becoming farther and farther removed from what would be considered optimal, or “healthy” cognitive aging.

This trend warrants attention from the perspective of cognitive aging research. Without adequate screening procedures in place, inclusion of individuals with T2DM and hypertension in otherwise healthy study samples may introduce unwanted variability and bias to brain and/or cognitive measures, and increase the potential for type 1 and type 2 errors. Functional neuroimaging studies may be particularly vulnerable in this regard. Blood oxygenation level dependent magnetic resonance imaging (BOLD MRI) and positron emission tomography (PET H_2_O^15^) measure hemodynamic changes associated with neural activity, and thus provide an indirect measure of neural function (Logothetis et al., 2001). To interpret individual or group differences in BOLD or PET signaling as reflective of individual or group differences in neural activation underlying a cognitive operation of interest, we rely on assumptions of intact neurovascular signaling, cerebrovascular reactivity, and vascular health amongst the study participants. These assumptions may be true in young and healthy individuals, but do not hold in older adults with conditions that affect vascular health (D'Esposito et al., 2003). Even normal, age-related changes in the integrity of the cerebrovascular system can undermine these assumptions (D'Esposito et al., 1999).

Yet, it was our impression that relatively few studies in the cognitive aging neuroimaging literature consider T2DM or hypertension during recruitment, or control for potential confounds associated with these conditions during analysis. To clarify the extent to which current research practices consider T2DM and hypertension in study design, we present the results of a systematic review of the cognitive aging neuroimaging literature, looking at study inclusion/exclusion criteria and methodology related to T2DM and hypertension. Then, to reinforce the notion that individuals with T2DM and hypertension should not be overlooked as a potential source of bias, we provide an overview of metabolic and vascular changes associated with T2DM and hypertension, as they relate to vascular health, structural brain atrophy, and functional integrity. The final section discusses best practices moving forward.

## Systematic review

This review focuses on the cognitive aging neuroimaging literature, however the issues associated with inclusion of individuals with T2DM and hypertension in study samples are by no means limited to this area of research. Any research study whose population of interest has high prevalence rates of T2DM or hypertension should be cognizant of these issues. For example, psychiatric populations have a higher incidence of metabolic disruption and T2DM that is mediated, at least partially, by the use of mood stabilizers, anticonvulsants, and antipsychotic medications (Regenold et al., 2002; Newcomer and Haupt, 2006).

It should also be noted that the purpose of this review is not to quantitatively compare the results of studies that have excluded T2DM and/or hypertension with those that have not. This type of comparison is not feasible for numerous reasons, the primary one being that the extent to which individuals with T2DM or hypertension were present in study samples that did not screen for either condition is unknown. Rather, the aim of this review is to highlight *the proportion* of studies in the cognitive aging neuroimaging literature that consider T2DM and/or hypertension in their inclusion/exclusion criteria, or attempt to account for the potential bias introduced by inclusion of these individuals in their study groups.

### Methods

**Figure 1:**
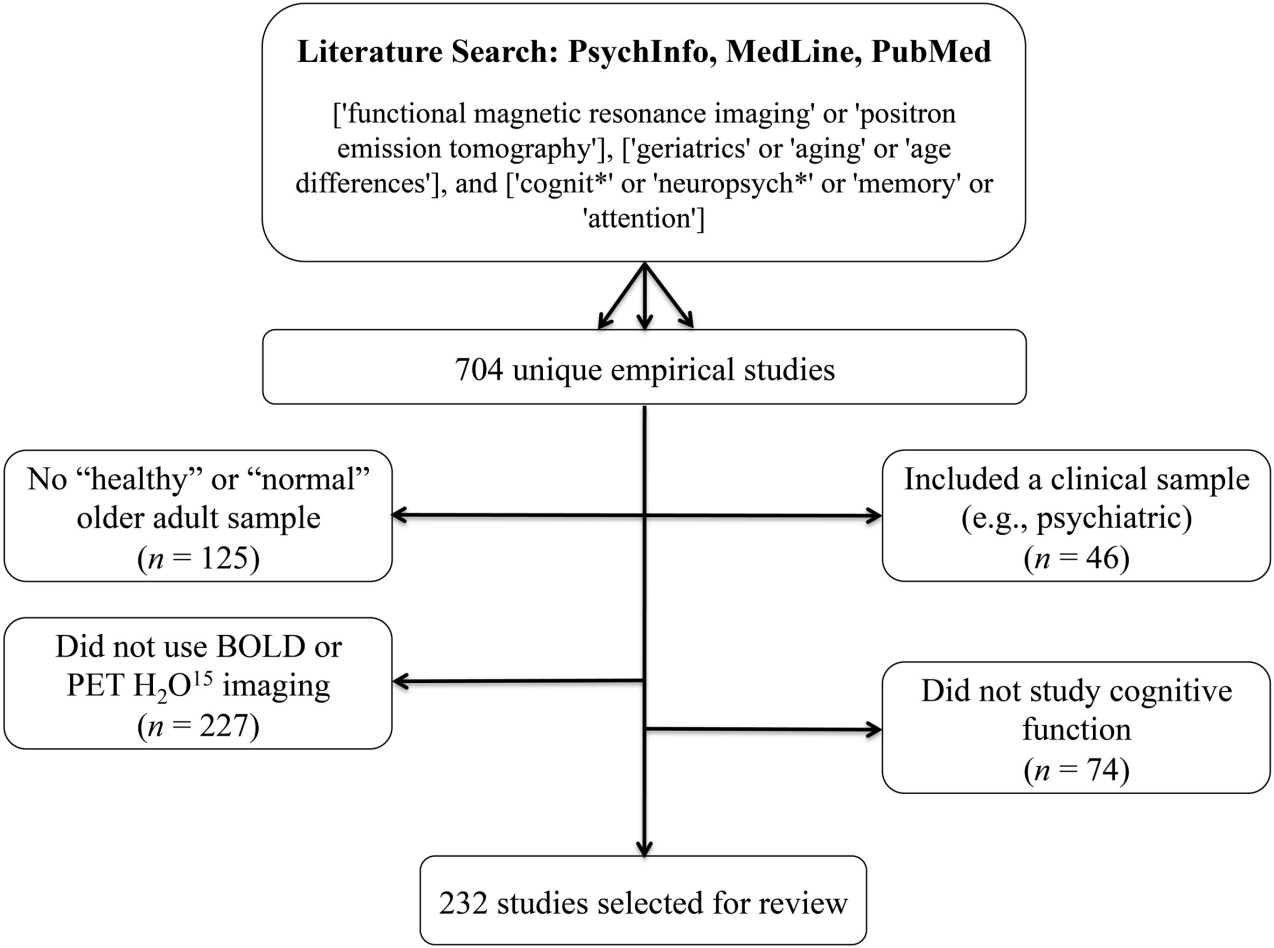
**Literature search terms and exclusion criteria**. Based on these criteria, 232 studies were selected for review.

We searched PsychInfo, MedLine, and PubMed between 1995 and February, 2013 using the search terms [“functional magnetic resonance imaging” or “positron emission tomography”], [“geriatrics” or “aging” or “age differences”], and [“cognit^*^” or “neuropsych^*^” or “memory” or “attention”]. Across the three databases, these search terms produced 704 unique empirical studies. From these results, we excluded studies that did not include a “healthy” or “normal” older adult sample (*n* = 125), included a clinical sample other than MCI or Alzheimer disease (AD)/dementia (e.g., psychiatric; *n* = 46), did not use BOLD or PET H_2_O^15^ imaging (*n* = 227), and did not scan during a cognitive or resting state task (*n* = 74; Figure 1).

### Results

Based on these criteria, 232 studies were selected for review. These studies are identified with an asterisk (^*^) in the reference section. Two hundred and nineteen of these used BOLD imaging, one used both BOLD and PET H_2_O^15^, and 12 used PET H_2_O^15^ only. One hundred and sixty five of these studies compared a “healthy” older group with a group of young participants, 34 studies compared a “healthy” older sample to an MCI and/or AD group (two of which also included a young adult comparison group), and the remaining 33 studies looked only at a “healthy” older sample. The majority of surveyed studies employed a memory paradigm during imaging (e.g., encoding/recognition of words, pictures, scenes, faces, autobiographical memory, spatial memory, associative memory, implicit learning). Working memory and executive processes were also well-studied (e.g., cognitive control, inhibition, decision making, mental rotation, task-switching, attention, judgment, processing speed, naming, imagery, verb generation, fluency). We also included resting-state studies in the sample.

Our primary concern was how sample selection was reported to have occurred. In particular, we were interested to learn how many studies specifically screened for T2DM and/or hypertension in their healthy older adult samples. For each of the 232 identified studies, the inclusion/exclusion criteria were examined according to the following criteria: (i) explicit exclusion of T2DM and/or hypertension, or exclusion of medical disorders/physical illnesses/systemic illnesses (implying that *all* medical conditions, including T2DM and hypertension, were excluded); (ii) exclusion of “significant,” “major,” or “severe” medical/physical/systemic disorders; or (iii) no screening criteria related to T2DM and/or hypertension provided. We also surveyed each of the 232 studies to determine how subjects were screened (e.g., self-report questionnaire, clinical assessment with a medical doctor, laboratory testing), and how—if at all—the potential influence of intersubject vascular variability on the measured BOLD or PET signals was addressed.

In each section below, superscript numbers, letters, and symbols are used to represent the extent to which studies screened for T2DM and hypertension, the screening method, and the degree to which studies attempted to account for intersubject vascular variability, respectively. The identified studies are denoted in the reference section according to these superscript classifiers.

#### Inclusion/exclusion of T2DM and hypertension

Of the 232 studies surveyed, only 22 (9.5%) explicitly excluded individuals with T2DM(^1^), and only 29 (12.5%) explicitly excluded individuals with hypertension(^2^). Thirteen studies—approximately 6%—excluded both T2DM and hypertension. Fourteen studies (6.0%) excluded individuals on antihypertensive medication(^3^), however few of these studies also clarified whether individuals were assessed for untreated hypertension and excluded, if present. Nineteen studies (8.2%) excluded medical illnesses, systemic illnesses, medical disorders or physical illnesses(^4^). This criterion implies that *all* medical conditions, including T2DM and hypertension, were excluded.

In contrast, almost half of the included studies (102; 44.0%) had exclusion criteria that made it difficult to determine whether T2DM was excluded(^5^), and 85 studies (36.6%) had exclusion criteria that made it difficult to determine whether hypertension and/or antihypertensive medications were excluded(^6^). These studies listed “major medical illnesses,” “significant medical conditions,” “serious systemic illnesses,” “conditions/medications interfering with cognitive and/or brain function,” “vascular disease,” “cardiovascular disease,” and/or “conditions/medications interfering with the fMRI signal” as exclusion criteria, or simply described their sample as “healthy.” There were also many studies that did not mention any selection criteria related to T2DM (80; 34.5%)(^7^) or hypertension (51; 22.0%)(^8^).

**Figure 2:**
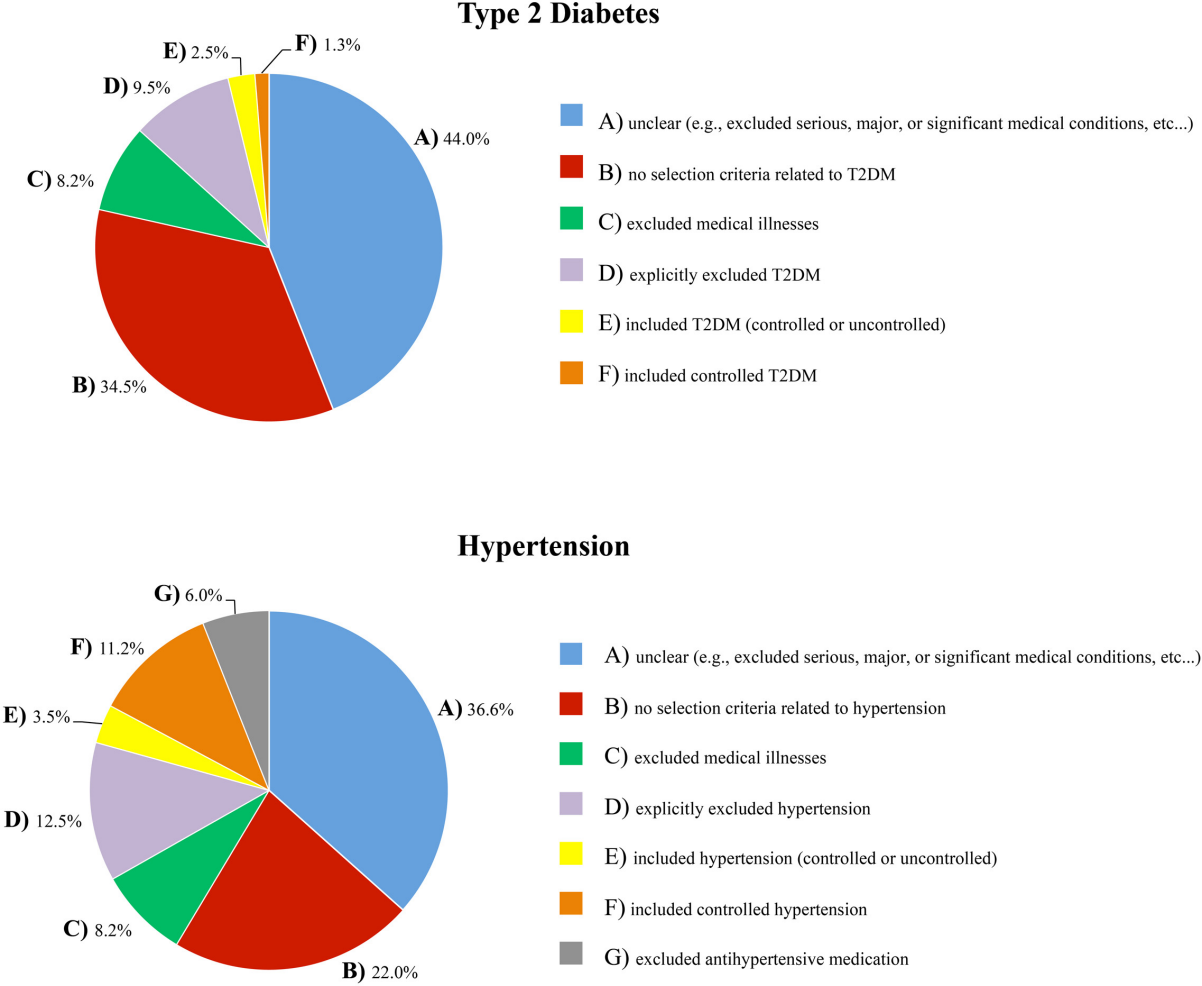
**The extent to which T2DM and hypertension were accounted for in the inclusion/exclusion criteria of the healthy samples that were surveyed**.

In addition, 26 studies (11.2%) included individuals with controlled hypertension(^9^), 8 studies (3.5%) included controlled *and* uncontrolled hypertension(^10^), 3 studies (1.3%) included individuals with controlled T2DM(^11^), and 6 studies (2.5%) included individuals with controlled *and* uncontrolled T2DM in their healthy cohort(^12^). Figure 2 provides a visual depiction of these results.

#### Screening method

The majority of studies (173; 75%) did not report how they conducted their medical screening(^a^). Only 28 studies (12%) reported having screened subjects with physician-conducted medical examinations and/or laboratory testing(^b^). Sixteen studies (7%) screened participants with telephone interviews, in-person clinical interviews, medical history, chart reviews, or a combination of these methods(^c^). The remaining 15 studies (6%) used a self-report questionnaire to assess medical status(^d^).

#### Accounting for intersubject vascular variability

A survey of the 232 included studies found that just under one third (29%) acknowledged and/or addressed the potential influence of intersubject vascular variability on the reported results. Many excluded subjects with a high vascular burden by screening for white matter hyperintensities in the imaging data(^■^). Others compared groups on vascular risk factors(^**+**^), compared outcome measures on hypertension status or antihypertensive treatment status(^♦^), or attempted to control for health, blood pressure, and/or white matter hyperintensities in the reported associations (^❖^). Several studies noted in their discussion the possibility that the reported results were influenced by vascular factors, or explained why they did not think this was an issue(^•^). A few studies used the measured BOLD or PET signals to examine and account for individual differences in vascular health(^□^); for example, by ensuring that groups were equated on BOLD signal variability, by comparing the temporal characteristics of the hemodynamic response curve across groups, with proportional scaling of the BOLD or PET signal, or by focusing on group by task interactions (instead of group main effects) or comparing within-subject task contrasts across individuals or groups to minimize any individual or group differences in vascular integrity.

There are rigorous ways to account for intersubject vascular variability, such as additional task data or an additional imaging contrast. Several studies included in the present review used arterial spin labeling (ASL) MRI (^▴^) or PET (^▾^) to measure resting cerebral blood flow and control for individual differences in perfusion. Three studies used a breath-hold task to index individual differences in cerebrovascular reactivity (^❍^), and two studies included a low-level motor or baseline task to ensure that participants demonstrated an adequate hemodynamic response (^**×**^).

### Discussion

Our results found that fewer than 10% of the selected functional imaging studies on cognitive aging explicitly excluded individuals with T2DM from their normative samples, and fewer than 15% explicitly excluded individuals with hypertension. A number of studies reported selection criteria that were insufficient to determine whether T2DM or hypertension were screened. Critically, one third of included studies had no reported inclusion or exclusion criteria related to T2DM, while almost a quarter had no reported inclusion or exclusion criteria related to hypertension. Only 67 of the 232 selected studies (29%) acknowledged or addressed the potential influence of intersubject vascular variability on the measured BOLD or PET signals.

Moreover, the large majority of studies did not include information about the medical screening process itself (e.g., laboratory testing vs. clinical interview vs. self-report questionnaire). This is not ideal when established tests for T2DM and hypertension are available (for example, 24-h ambulatory blood pressure monitoring would be the gold-standard for determining hypertension status, and an oral glucose tolerance test for determining T2DM status). Furthermore, we posit that participants may be less likely to volunteer T2DM or hypertension status as a “significant” medical illness without specific probing (i.e., compared to cancer, HIV, multiple sclerosis, or heart disease), because when these conditions are well-controlled they can have a minimal impact on day-to-day functioning, and, in the case of T2DM, can be controlled by diet alone. Collectively, these observations point to a lack of awareness that T2DM and hypertension are major medical illnesses that interfere significantly with cognitive and brain function in older adults.

## Overview: metabolic and vascular complications of type 2 diabetes mellitus and hypertension

To reinforce the position that T2DM and hypertension are conditions that can have a major effect on brain health and cognitive aging, this next section reviews evidence on the cognitive deficits, structural changes, and functional consequences associated with T2DM and hypertension, and describes some of the mechanisms that mediate these changes.

### Type 2 diabetes mellitus

T2DM is the result of peripheral insulin resistance, which leads to insulin dysregulation and hyperglycemia. These metabolic changes affect cerebrovascular health, structural integrity, and brain function, and underlie the associations between T2DM, cognitive decline, and dementia risk.

#### Insulin dysregulation

Insulin is a peptide hormone that is critical for regulation of blood glucose levels. Binding of insulin to its receptors, found on nearly all cells throughout the body, facilitates the cellular uptake of glucose from the blood. When bound, insulin and insulin-like growth factor also activate complex intracellular signaling pathways that promote cell growth and survival, regulate glucose metabolism, and inhibit oxidative stress and apoptosis (for a review, see Nakae et al., 2001).

The defining characteristic of T2DM is peripheral insulin resistance, which occurs when cells in the body decrease their response to insulin stimulation. In the developing stage of this disease, the pancreas is able to produce enough insulin to overcome this resistance. This results in peripheral hyperinsulinemia, and blood glucose levels remain within the normal range. As the disease progresses, however, the pancreas can no longer keep up, and blood glucose levels begin to rise. When blood glucose levels are high even in the fasting state, T2DM is diagnosed.

Peripheral insulin resistance and hyperinsulinemia have a counterintuitive impact on insulin levels within the central nervous system. In the face of peripheral hyperinsulinemia, insulin transport across the blood brain barrier is effectively reduced, resulting in a brain *hypo*-insulinemic state (e.g., Heni et al., 2013). Low brain insulin levels and disrupted insulin signaling contribute to cognitive impairments directly, particularly in medial temporal lobe regions where insulin receptors are abundant (Convit, 2005; Craft, 2006). Indirectly, low brain insulin levels exacerbate amyloid beta (Aβ) and tau pathology, hallmarks of Alzheimer disease (AD). It is here that we see the link between T2DM and Alzheimer disease pathology: brain insulin deficiency results in the down-regulation of insulin degrading enzyme (IDE; Luchsinger, 2008), which also has a role in degrading Aβ (Carlsson, 2010). As a result, Aβ degradation is effectively reduced, contributing to its aggregation and amyloid plaque formation. Decreased brain insulin levels also suppress the enzymes involved in tau phosphorylation, contributing to the formation of neurofibrillary tangles (Akter et al., 2011). While the downstream impact of T2DM-mediated brain insulin deficiency and insulin resistance is more moderate than that associated with AD, the underlying pathogenic mechanisms are similar (Steen et al., 2005), and it has been proposed that AD is a form of diabetes mellitus that selectively affects the brain (T3DM; for discussion, see de la Monte and Wands, 2008). Given this, is not surprising that individuals with T2DM show a pattern of memory impairment, medial temporal lobe atrophy, and reduced hippocampal connectivity that is similar to the classic pattern of memory deficits, neurodegeneration, and network disruption in AD (e.g., Gold et al., 2007; Zhou et al., 2010; Baker et al., 2011; Cui et al., 2014).

#### Hyperglycemia

When cells in the body become resistant to the effects of insulin, blood glucose levels rise, resulting in hyperglycemia. Endothelial cells are particularly vulnerable to the effects of hyperglycemia, because they are less efficient at reducing glucose uptake in the face of high blood glucose levels (Kaiser et al., 1993). Under such conditions, the resultant *intracellular* hyperglycemia induces an overproduction of reactive oxygen species in the mitochondria, which increases oxidative stress within the cell. This initiates a cascade of biochemical events that mediate much of the microvascular and macrovascular damage associated with T2DM including, but not limited to, increased intracellular formation of advanced glycation end-products (AGEs) and protein kinase C activation (Du et al., 2000; Nishikawa et al., 2000; Brownlee, 2005; Giacco and Brownlee, 2010; Johnson, 2012).

AGEs are formed during normal metabolism on proteins with slower rates of turnover, in almost all cells throughout the body. AGE accumulation over time is a major factor in normal aging; however, under hyperglycemic conditions, AGE production is exacerbated beyond normal levels. AGEs cause intracellular damage and induce apoptosis through a process called cross-linking (Shaikh and Nicholson, 2008). AGEs also contribute to oxidative stress, and themselves activate inflammatory signaling cascades (for a review, see Yan et al., 2008). Critically, under hyperglycemic conditions, the Aβ protein itself can act as an AGE (Granic et al., 2009), which enhances its own aggregation and further increases amyloid plaque formation.

Protein kinase C activation, on the other hand, affects a variety of changes in gene expression that culminate in vascular dysfunction. Production of nitric oxide (NO), a vasodilator, is decreased, and production of endothelin-1, a vasoconstrictor, is increased. As a result, blood vessels are less able to dilate to accommodate increased blood flow demand. Over time, chronic exposure to high concentrations of endothelin-1 and decreased concentrations of NO contribute to diminished vessel elasticity, and structural changes in the vessel wall that result in atherosclerotic plaque formation (Kalani, 2008).

In the brain, hyperglycemia-mediated macro- and microvascular damage reduces the delivery of nutrients and oxygen required to meet metabolic demands. Altered cerebral autoregulation has been observed in middle-aged adults with T2DM (Brown et al., 2008), and may be an early manifestation of microvascular disease (Kim et al., 2008). Older adults with T2DM show decreased blood flow velocity, increased cerebrovascular resistance, and impaired vasoreactivity (Novak et al., 2006). Over time, declines in cerebrovascular health and reduced perfusion of brain tissue lead to structural atrophy and altered brain function.

#### Cognitive effects

The cognitive profile of individuals with T2DM includes deficits in attention, processing speed, learning and memory, and executive function (e.g., Reaven et al., 1990; Brands et al., 2007; Yeung et al., 2009; Whitehead et al., 2011). Moreover, these individuals, and individuals with pre-diabetes (impaired glucose tolerance), show an accelerated trajectory of cognitive decline relative to that associated with healthy aging (Gregg et al., 2000; Fontbonne et al., 2001; Arvanitakis et al., 2004; Yaffe et al., 2004; Fischer et al., 2009; Nooyens et al., 2010; Espeland et al., 2011; for conflicting results, see van den Berg et al., 2010).

Cognitive deficits in T2DM have been linked to multiple disease-related processes, including: (i) poor glucose control (i.e., hemoglobin A1c [HbA1c]; Ryan and Geckle, 2000; Kanaya et al., 2004; Cukierman-Yaffe et al., 2009; Maggi et al., 2009; Luchsinger et al., 2011; Tuligenga et al., 2014; for conflicting results, see Christman et al., 2011), (ii) glucose intolerance (Rizzo et al., 2010; Zhong et al., 2012b), (iii) high peripheral AGE levels (Yaffe et al., 2011), (iv) high levels of inflammatory cytokines (Marioni et al., 2010), and (v) peripheral hyperinsulinemia and insulin resistance (Bruehl et al., 2010; Zhong et al., 2012a). Even in non-diabetic adults, poorer glucoregulation has been associated with deficits and/or declines in verbal memory, working memory, processing speed, and executive function (Dahle et al., 2009; Bruehl et al., 2010; Messier et al., 2010, 2011; Ravona-Springer et al., 2012).

The link between cognitive impairment and poor metabolic control may be largely mediated by the structural and functional brain changes that occur in the presence of chronic insulin dysregulation and hyperglycemia. Associations between glucoregulation, hypoperfusion in temporal regions, hippocampal atrophy, and memory impairment have been observed in T2DM (Gold et al., 2007; Last et al., 2007), and in non-diabetic adults with decreased peripheral glucose regulation (Convit et al., 2003), or high fasting plasma glucose levels within the normal range (Cherbuin et al., 2012; Kerti et al., 2013). In other studies of T2DM, cognitive deficits and structural brain atrophy were linked to cerebral hypoperfusion and altered vascular reactivity (Last et al., 2007; Brundel et al., 2012), and disrupted default-mode network connectivity was associated with peripheral hyperinsulinemia, insulin resistance, and white matter integrity (Musen et al., 2012; Hoogenboom et al., 2014). Regardless of the underlying cause, brain atrophy in T2DM is associated with poor cognition (Moran et al., 2013), and cognitive declines have been associated with progression of brain atrophy over time (van Elderen et al., 2010; Reijmer et al., 2011). Some studies suggest that structural changes may occur early in the course of T2DM; enlarged lateral ventricles, particularly within the frontal horns, have been observed less than a year after diagnosis (Lee et al., 2013), and middle-aged, as well as older adults with T2DM, show reduced prefrontal volumes (Bruehl et al., 2009) and generalized global atrophy (de Bresser et al., 2010; Kamiyama et al., 2010; Espeland et al., 2013).

### Hypertension

The brain is one of the most highly perfused organs. The cerebral hemispheres are supplied by capillary beds connected to the pial vasculature by penetrating arterioles, and the pial vasculature stems from a system of arteries branching off the anterior, middle, and posterior cerebral arteries. Maintenance of brain function depends on a constant blood supply through this network. Hypertension causes changes to the structure and function of these blood vessels, which impacts perfusion in affected areas. Hypoperfusion, for example, can interfere with the delivery of oxygen and nutrients required to meet metabolic demands, and makes hypertension a major risk factor for vascular cognitive impairment, stroke, and dementia.

#### Cerebrovascular changes

Hypertension places enormous stress on the cerebral circulation (for a comprehensive review, see Pires et al., 2013). A hallmark of chronic hypertension is increased vascular resistance, particularly in the small blood vessels that perfuse the brain. Vascular resistance increases as vessel walls thicken. This remodeling is an adaptive response required to maintain chronically increased blood pressure, but it decreases the interior space of the blood vessels (the lumen). Vascular resistance also increases as the number of blood vessels decrease. Rat models of hypertension have shown both of these effects: reductions in lumen diameter and in the number of capillaries making up capillary beds in the cerebral vasculature (Sokolova et al., 1985).

Blood flow is reduced when vascular resistance is high, and chronic hypertension-mediated hypoperfusion has been linked to white matter degradation, gray matter atrophy, and cognitive deficits. Studies of older adults with hypertension show reduced blood flow, particularly in occipito-temporal, prefrontal, and medial temporal lobe regions (Beason-Held et al., 2007), positive correlations between blood pressure and white matter burden (White et al., 2011; Raji et al., 2012), and negative correlations between blood pressure and total brain volume (Nagai et al., 2008). Blood vessel function is also impacted by hypertension. Cerebral autoregulation (i.e., the ability to maintain a constant perfusion rate over a range of arterial pressures) is impaired, as is cerebrovascular reactivity, the ability of blood vessels to dilate to accommodate increased blood flow demand (Last et al., 2007; Hajjar et al., 2010).

#### Cognitive effects

The cognitive profile of older adults with hypertension includes poorer performance on tests of executive function, including verbal fluency, Trails B-A switching score, Stroop interference scores (Bucur and Madden, 2010), slowed processing speed (Dahle et al., 2009), and deficits in attention and memory (see Gifford et al., 2013 for a meta-analysis). Prospective cohort studies show that midlife cardiovascular risk factors like hypertension predict cognitive impairment in later life (e.g., Virta et al., 2013), and, similarly, cross-sectional studies show a relation between higher systolic blood pressure and poorer cognitive performance, even within the normotensive range, a relation that is particularly strong in midlife (e.g., Knecht et al., 2008, 2009). Hypertension is associated with decreases in cognitive reserve (Giordano et al., 2012), and older adults with MCI and cardiovascular risk factors like hypertension are twice as likely to develop dementia compared to those without such risk factors (Johnson et al., 2010; Ettorre et al., 2012). Moreover, cognitive declines may be faster in those with MCI and hypertension, compared to those without hypertension (Li et al., 2011; Goldstein et al., 2013).

The association between hypertension and cognitive decline appears to be strongest in executive and processing speed domains, and weakest in memory and language domains. Hypertension increased the risk of non-amnestic MCI, but not amnestic MCI, regardless of APOEε 4 genotype or hypertensive medication status (Reitz et al., 2007), and predicted progression to dementia in non-amnestic MCI, but not amnestic or multi-domain MCI (Oveisgharan and Hachinski, 2010). The impact of hypertension on executive and processing speed domains is consistent with studies that show a positive relation between hypertension and white matter changes (Kennedy and Raz, 2009; Raz et al., 2012), and between white matter changes and deficits in processing speed, executive function, and attention, but not memory (e.g., Debette et al., 2011).

Cognitive deficits in hypertensive adults are linked to various indicators of vascular and brain health. There are correlations between white matter integrity and performance on tests of executive function and attention (Hannesdottir et al., 2009), and between decreased flow-mediated dilation and poorer executive function (Smith et al., 2011). Deficits in attention and psychomotor speed in late middle-aged adults with hypertension are associated with reductions in global brain perfusion, reductions that were not fully ameliorated following 6-months of antihypertensive treatment (Efimova et al., 2008). Global cognitive decline has been linked to reduced cerebral blood flow in the face of white matter lesions and lacunar infarcts (Kitagawa et al., 2009), to higher pulse pressure and arterial stiffness (Scuteri et al., 2007; Waldstein et al., 2008; Triantafyllidi et al., 2009), and to hypertension-mediated deep-brain vascular pathology (Yakushiji et al., 2012). In another large study of patients with MCI, those with hypertension and deep white matter lesions were at higher risk of dementia (Clerici et al., 2012).

## Conclusions

Taken together, these studies provide abundant evidence that middle-aged and older adults with T2DM and hypertension, relative to healthy older adults, are more likely to show signs of cognitive dysfunction, widespread structural atrophy, vascular damage, and functional changes. In light of their rising prevalence amongst older adults, there is an increasing likelihood that, without adequate screening at recruitment, individuals with T2DM and/or hypertension will be included in healthy older adult samples. This may introduce unwanted variability and bias to brain and/or cognitive measures, and increase the potential for type 1 and type 2 errors. Given the state of the neuroimaging literature on this topic and the need to advance our understanding, we view T2DM and hypertension as important new frontiers in cognitive neuroscience.

Moving forward, there is an opportunity to develop best practices when it comes to cognitive neuroscience research in older adult populations. Reconciling the vascular risk component in T2DM and hypertension may be the most tractable option since there are myriad approaches one can take to do this. The most rigorous approach in this respect may be inclusion of a breath-hold task, or a measure of cerebral blood flow (e.g., ASL) in the functional imaging protocol, as this allows for a direct estimate of each subject's vascular health. Breath-hold tasks can be used to index cerebrovascular reactivity in response to non-neuronal signals. The breath-hold period induces hypercapnia, which stimulates vasodilation and increases blood flow and blood volume in the brain, a signal change that occurs independently of neuronal activation. ASL or resting-state PET scans provide a direct measure of blood flow, and can be used to account for individual differences in perfusion. As noted above, these methods have already been used in some studies of cognitive aging to account for individual differences in cerebrovascular health. Whether other means of equating vascular risk across participants or across groups (e.g., screening participants for excessive white matter hyperintensities, *post-hoc* comparison of outcome measures or study groups on vascular risk factors, or statistical analyses aimed at controlling for the effects of vascular variability in the reported results) are similarly effective requires further study.

It may also be important for investigators to acknowledge a distinction between “healthy” and “typical” brain aging. Studies characterizing *healthy* aging should adopt T2DM and hypertension as exclusion criteria. Conversely, given the high prevalence of T2DM and hypertension in older adults, community- or population-based studies characterizing the *typical* trajectory of cognitive aging would benefit by including these participants to maximize the generalizability of results, and reconciling the heterogeneity through study design groups (e.g., stratifying based on diagnosis of T2DM and hypertension) or covariates in their analysis.

As the proportion of older adults living with T2DM and hypertension increase, it is imperative that functional imaging studies are designed to account for these population trends. The current state of the cognitive aging neuroimaging literature suggests that there is limited appreciation and/or awareness that T2DM and hypertension are significant medical illnesses that disrupt brain vasculature, brain structure, and brain function. By adopting best practices that take T2DM and hypertension into account, we can advance our understanding of these conditions, and of cognitive aging in general.

## Author contributions

Liesel-Ann C. Meusel selecting, indexing, and reviewing articles, writing of drafts; Nisha Kansal selecting articles, editing of drafts; Ekaterina Tchistiakova contributing to the first draft, editing of drafts; William Yuen selecting articles, contributing to the first draft, editing of drafts; Bradley J. MacIntosh provided conceptual foundation for paper, editing of drafts; Carol E. Greenwood provided conceptual foundation for paper, editing of drafts; Nicole D. Anderson provided conceptual foundation for paper, editing of drafts.

### Conflict of interest statement

The authors declare that the research was conducted in the absence of any commercial or financial relationships that could be construed as a potential conflict of interest.
